# Transactions of Amer. Med. Ass’n.

**Published:** 1871-12

**Authors:** 


### BOOK REVIEWS.

Transactions of the American Medical Association, vol. 22., 1871.

This number, although somewhat smaller than usual, is fully equal in the value and importance of the report contained, to any previous volume of the transactions of the association.

In the report of F. W. Hatch, M. D., on the "Climatology and Epidemics of California," a number of new and interesting facts are brought forward, especially in regard to the relation of that climate to consumption. Very full and valuable tables are given, showing the temperature and humidity throughout the year at Sacramento, and also at Darmer's Pass (Summit) Sierra Nevada, at an elevation of 7091 feet.

In table C, the deaths from diseases of the respiratory class at San Francisco and at Sacramento, for the year 1870 are given, the deaths from consumption being included. In San Francisco they were as follows: from asthma, 5; bronchitis, 38; laryngitis, 9; congestion of the lungs, 35; inflammation of the lungs, 196; hemmorrhage of the lungs, 18; other diseases of this order, 28; consumption, 495. To Sacramento, the table assigns deaths from asthma, 1; bronchitis, 2; inflammation of the lungs, 26; other diseases of this order, 5; consumption, 79. The writer, however, says: "It should be borne in mind that to both of these localities invalids are continually brought for private and hospital attendance, thus swelling the death-rate beyond its legitimate proportions. This is especially true of phthisic."

As a summer resort for the consumptive invalid, the author considers that no better or more salutary location could be selected than the elevated mountain ranges of the Sierra Nevada, or the east side of the coast range mountains. In both of these mountain ranges many distinct localities are now becoming places of frequent resort both for the invalid and the seeker after pleasure. "The atmosphere is dry and invigorating; the water is pure and cold; the streams abound in trout and the hills in game; while in the valleys, lying here and there between separate ridges or spars of the mountains, every desirable comfort can be obtained. In the Sierra Nevada range, we have Lake Valley lying at the base of one of the great ridges of the range, on an elevation of about 7000 feet, inclosing the largest of our mountain lakes—'Lake Bigler,'—a sheet of water about forty miles long, and probably ten wide. Then there is Hope Valley, a small but attractive plain just over the summit, and watered by the head of Carson River, where the invalid may find, during the summer months, a climate delightfully adapted to physical comfort, dry and bracing, affording in the clear stream which courses near its centre the best possible facilities for enjoyment to those fond of fishing; then there is, a few miles distant, Silver Lake, a beautiful sheet of water, one mile and a half long by one mile wide, set like a polished mirror in the basin of a deep depression between surrounding hills, perhaps 2000 feet below their crests, and itself 2000 feet above the sea, displaying in its steep approach and in its immediate surroundings one of the grandest views which nature in her extravagance of scenery has afforded. These, and other localities which might be enumerated, may be regarded as supplying every possible advantage to be found in our mountain climates."

"This much for a summer residence. Yet even in the winter season, it is questionably whether the climate of some portions of the mountains would not be equally beneficial for a very large class of invalids. The air, although cold, is still dry in comparison with that of the valleys, and it remains to be proved that mere cold, without excessive and continued moisture, is specially injurious to those suffering with the early stages of consumption. On the east the ranges of the Sierra Nevada are for a greater portion of the season covered with snow, often for a great depth, and, except on the principal routes of travel, communication is difficult. The opening of the Pacific Railroad, however, has latterly afforded facilities for intercourse which were previously unknown, and has thus obviated one important obsticle, hitherto insuperable.

The same objection does not exist in regard to the coast range. Here snow, except on the higher crests, is not frequently seen, never for any very considerable duration, and the atmosphere for the greater portion of the winter is cool, dry, and bracing. Relying, however, upon our own experience and observation, we are inclined to give the preference, for the winter season, to some of the valleys in the extreme southern portion of the State, as for example, a residence in San Diego. In this delightful section the rainy season is of shorter duration than in the more northern valleys, the climate is warmer and more equable, and the atmosphere drier. Of a somewhat similar climatic character is the neighborhood of Santa Borbora, situated a short distance from the ocean, about 200 miles, in a direct line, south of San Francisco, in one of the most attractive of the coast valleys, a region of country excelling in natural advantages, and to which the attention of immigration is being directed on account of its equable and salubrious climate, and the moderateness of its winter temperature, the fertility of its soil, and its adaptibility to the most varied and profitable culture."

These quotations from the report of Dr. Hatch embody in the main, his conclusions in regard to the effects of the climate of different portions of California upon consumption.

The report on the climatology and epidemics of Minnesota, by Charles N. Hewett, M. D., also evinces a good deal of care and labor in its preperation, and forms quite a complete exposition of the subject. The subject of phthisic pulmonalis is of especial interest in this connection, and in regard to it the writer reports as follows:

"Origin in the State.—In the reports received, 52 cases are given which originated in the State: with known predispositions, 8 cases, without known predispositions, 9 cases.

"Dr. Senkler, of St. Cloud, of 30 cases examined, reports 20 per cent originating here, all with predispositions.

"Effects of Residence on Incipient Disease.—Drs. Willey, Hand, Murphy, C. E. Smith and Mattocks, old residents of St. Paul, the headquarters for most of this class of patients from abroad, report it very favorable. Dr. Murphy thinks in 90 per cent of cases. Drs. Hawley, of Red Wing; Galloway, of Rochester; Adams, of Hastings, Hill, of Pine Island; Sheardown, of Stocton; Senkler, of St. Cloud; Griswold, of Taylor's Falls; Reiner and Rhodes, of Stillwater, report it favorable, Dr. Sweeney, of Red Wing, after a careful review of 21 years experience in the State, states his decided conviction that 90 per cent of cases in which the diagnosis is certain will die within two years of the disease. Dr. Staples, of Winona, makes two classes under this head. 1. Those with general debility and anæmia, dependent on poor digestion and faulty assimilation, these are benefited. Dr. Mays, of Rochester, agrees with this statement. 2. In those cases in whom there is a conjestive tendency (a disposition to hæmoptysis,) the stimulant effect of the climate may be injurious."

In regard to the effects upon developed disease, the following report favorably: Drs. Willey and Hand, of St. Paul, if not complicated with bronchitis; Murphy, of St. Paul, if not of both lungs; Mattocks, of St. Paul, cases have been benefited, not advised; C. E. Smith, of St. Paul, permanent or partial relief; Mayo, of Rochester, end deferred; Adams, of Hastings, to prolong life.

Unfavorable.—Mattocks, of St. Paul, as also Staples, same rule as in incipient disease, not generally so favorable; Sweeney, of Red Wing, majority die in six months; Hawley and Hewett, of Red Wing, not favorable, end hastened.

In an appendix at the conclusion of the report, Dr. Hawley reports a number of cases of typhoid fever with a view of establishing and proving the contagious and infectious nature of the disease.

The report of J. P. Moore, M. D., on the climatology and epidemics of Mississippi, contains valuable and important information in regard to that region. The following are given as the most prevelent diseases of the State named in the order of their fatality:

Pneumonia, malarial fever, inflammations of the brain and membranes, mostly affecting children, typhoid fever, dysenterty, diarrhœa, consumption, now much on the increase, cholera infantum, irritative fever.

The following is a list of the remaining reports contained in the volume:

Report of Section on Practice of medicine and Obstetrics.

Report of Committee on Criminal Abortion.

Report of Section on Materia Medica and Chemistry.

Report of Section on Surgery and Anatomy.

A synopsis and analysis of one hundred cases of Lithotomy and Lithotrity, etc., by Paul F. Eve, M. D.

Improved Trocar for Ovariotomy and other purposes, by A. M. Pollock, M. D.

A New Observation on the Luxated Elbow, by A. S. Hudson, M. D.

Prize Essay on the Chemical Constitution of the Bile, by Edward R. Taylor, M. D.

Prize Essay—The Direct Method of Artificial Respiration for the treatment of persons apparently dead from suffocation by drowning or from other causes, with cases, by Benjamin Howard, M. D.

